# GCN5 modulates osteogenic differentiation of periodontal ligament stem cells through DKK1 acetylation in inflammatory microenvironment

**DOI:** 10.1038/srep26542

**Published:** 2016-05-24

**Authors:** Bei Li, Jin Sun, Zhiwei Dong, Peng Xue, Xiaoning He, Li Liao, Lin Yuan, Yan Jin

**Affiliations:** 1 State Key Laboratory of Military Stomatology, Center for Tissue Engineering, School of Stomatology, Fourth Military Medical University, Xi’an, Shaanxi 710032, China; 2 Research and Development Center for Tissue Engineering, Fourth Military Medical University, Xi’an, Shaanxi 710032, China; 3 Department of Stomatology, the First Affiliated Hospital of Guangzhou Medical University, Guangzhou, Guangdong 510140, China; 4 Department of Oral and Maxillofacial surgery, General Hospital of Shenyang Military Area Command, Shenyang, Liaoning 110840, China

## Abstract

Periodontal ligament stem cells (PDLSCs) from periodontitis patients showed defective osteogenic differentiation. However, the mechanism of impaired osteogenic differentiation of PDLSCs in inflammatory microenvironments is still unclear. In this study, we found that inflammation in the microenvironment resulted in downregulation of histone acetyltransferase GCN5 expression and lack of GCN5 caused decreased osteogenic differentiation of PDLSCs. Previous study showed activated Wnt/β-cateinin pathway of PDLSCs resulted in defective osteogenic differentiation. Here we found knockdown of GCN5 decreased the expression of DKK1, an inhibitor of Wnt/β-cateinin pathway, thus activated Wnt/β-catenin pathway of PDLSCs. Mechanistically, GCN5 regulated DKK1 expression by acetylation of Histone H3 lysine 9 (H3K9) and Histone H3 lysine 14 (H3K14) at its promoter region. Interestingly, we found that *in vivo* injection of aspirin rescued the periodontitis of rats through inhibiting inflammation and upregulating GCN5 expression. Furthermore, aspirin treatment of PDLSCs upregulated GCN5 expression and increased osteogenic differentiation of PDLSCs. In conclusion, GCN5 plays a protective role in periodontitis through acetylation of DKK1 and applying drugs targeting GCN5, such as aspirin, could be a new approach for periodontitis treatment.

Mesenchymal stem cells (MSCs) are multipotent progenitor cells capable of self-renew, multilineage differentiation, and immunomodulation^1^,^2^. MSCs maintain the bone homeostasis due to their multilineage differentiation potentials including osteogenesis, adipogenesis and chondrogenesis^3^,^4^. Microenvironment or the niche often governs the lineage commitment and cell differentiation of stem cells^5^. MSCs exhibited defective osteogenic differentiation in inflammatory microenvironments^6^,^7^,^8^. Periodontal ligament stem cells (PDLSCs) are a group of MSCs derived from periodontal ligament and defective osteogenic differentiation of PDLSCs were documented being closely related to periodontitis^9^,^10^. Besides, osteogenic deficit could not be recovered from *ex vivo* culture and expansion. Such deficiency of stem cells seem to retain a “memory” of abnormal microenvironment, which suggests the epigenetic modification may be involved. However, it remains unknown if epigenetic modification regulated the osteogenic differentiation of PDLSCs in inflammatory microenvironments.

The differentiation potential in MSCs often relies on orchestrated activation or repression of lineage specific genes. Previous studies have been focused on identifying numerous extrinsic regulators and their downstream transcription factors that control cell differentiation^11^,^12^,^13^. However, cell differentiation can be regulated more specifically at the epigenetic level^14^. Upon differentiation of MSCs, the epigenetic marks change to reflect the activation and repression of cell differentiation genes and modified histone domains have thus become epigenetic signatures that either mark for gene activation or repression^14^. Growing evidences suggest that inflammatory microenvironments change the posttranscriptional modification of histone by acetylation^15^,^16^,^17^. Acetylation of lysine residues entails the addition of an acetyl group on histone residues by histone acetyltransferases (HATs), thereby enabling the regulation of transcription. Additionally, multiple lines of evidence indicate HAT is associated with stem cell differentiation^18^,^19^.

GCN5 (General control non-repressed protein5), also named KAT2A, was one of the first HAT linked to gene transcriptional activation and regulates a chromatin program of histone acetylation that is required for normal embryogenesis^20^. Previous studies also showed that GCN5 played an important role in regulating differentiation of stem cells^21^,^22^. However, it is still unknown if GCN5 regulates the osteogenic differentiation of MSCs in inflammatory microenvironments. Aspirin is widely used for the treatment of inflammation and it is previous reported capable of elevating osteogenesis of MSCs^23^. Based on these reasons, we wonder if aspirin could be used to prevent periodontitis by regulating GCN5 expression and increasing the osteogenic differentiation of PDLSCs.

To further explore the role of GCN5 in osteogenic differentiation of MSCs in inflammatory microenvironment, using PDLSCs as a model, we compared the expression of GCN5 in PDLSCs from periodontally healthy subjects and periodontitis patients. In addition, we knockdown GCN5 in PDLSCs from periodontally healthy subjects and overexpressed GCN5 in PDLSCs from periodontitis patients to observe the osteogenic differentiation. We found that lack of GCN5 decreased the osteogenic differentiation of PDLSCs and overexpression of GCN5 rescued osteogenic deficiency in PDLSCs from periodontitis patients. Mechanistically, GCN5 regulated DKK1 expression by acetylation of Histone H3 lysine 9 (H3K9) and Histone H3 lysine 14 (H3K14) to regulate Wnt/βcatenin pathway of PDLSCs. Interestingly, we also found aspirin inhibited the formation of periodontitis through inhibiting inflammation and upregulating GCN5 expression in lipopolysaccharide (LPS)-induced periodontitis rats.

## Results

### Down-regulated expression of GCN5 in PDLSCs in inflammatory microenvironments

**Figure 1:**
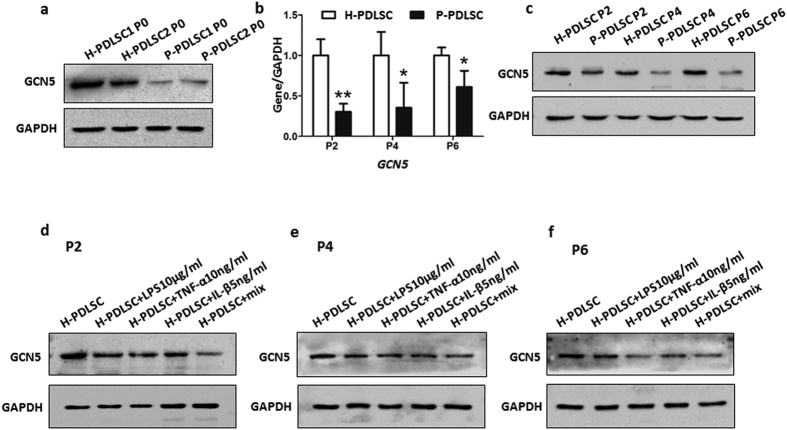
Down-regulated expression of GCN5 in PDLSCs in inflammatory microenvironments. (**a**) Western blot analysis showed the protein expression of GCN5 of freshly isolated H-PDLSCs and P-PDLSCs, and GAPDH was used as the internal control. (**b**) Relative gene expression of GCN5 of 2, 4, 6 passage H-PDLSCs and P-PDLSCs was analyzed by qRT-PCR. Relative gene expression of GCN5 was determined based on the threshold cycle (CT) values. The expression levels of the target genes were normalized to that of the housekeeping gene GAPDH. (**c**) Western blot analysis showed the protein expression of GCN5 of 2, 4, 6 passage H-PDLSCs and P-PDLSCs, and GAPDH was used as the internal control. (**d**) Western blot analysis showed the protein expression of GCN5 of 2 passage H-PDLSCs after either alone or in combination of LPS, TNF-α and IL-1β treatment. (**e,f**) Western blot analysis showed the protein expression of GCN5 of 4 and 6 passage H-PDLSCs, which were passed by 2 passage H-PDLSCs treated with either alone or in combination of LPS, TNF-α and IL-1β. GAPDH was used as the internal control. Data represent the means ± SD. *p < 0.05, **p < 0.01, (n = 3).

To know if GCN5 was differently expressed in PDLSCs from periodontally healthy subjects (H-PDLSCs) and periodontitis patients (P-PDLSCs), we first isolated H-PDLSCs and P-PDLSCs and characterized them by flow cytometry analysis. Both H-PDLSCs and P-PDLSCs expressed the surface markers of MSCs (Supplementary Fig. S1). Then PCR and western blot analysis were performed to measure the expression of GCN5 in gene and protein level of H-PDLSCs and P-PDLSCs. Western blot analysis showed that lower expression of GCN5 was decreased in P-PDLSCs derived from two patients compared to H-PDLSCs from two periodontally healthy subjects (Fig. 1a). The results showed that lower expression of GCN5 in passage 2, 4, 6 of P-PDLSCs compared to the same passage of H-PDLSCs both in gene (Fig. 1b) and protein (Fig. 1c) level, which indicated that GCN5 was downregulated in P-PDLSCs and the results of expression level were consistent after culture *in vitro* for different passage.

Periodontitis is an inflammatory disease in which higher expression of TNF-α, IL-1β and LPS were found in the periodontal microenvironment in patients^24^. To mimic the inflammatory microenvironment, we respectively used LPS (10 μg/ml), TNF-α (10 ng/ml) and IL-1β (5 ng/ml) alone or in combination to treat H-PDLSCs of passage 2. After either alone or in combination of LPS, TNF-α and IL-1β treated, expression of GCN5 was down-regulated compared to H-PDLSCs without treatment (Fig. 1c). Then we passed the cells to passage 4 and passage 6, expression of GCN5 still was down-regulated in LPS, TNF-a and IL-1β and mixed factors groups (Fig. 1d,e) by western blot assay. These results suggested that inflammatory microenvironment decreased GCN5 expression of PDLSCs and might result in the epigenetic modification of PDLSCs.

### Down-regulated GCN5 expression results in defective osteogenic differentiation of P-PDLSCs

**Figure 2:**
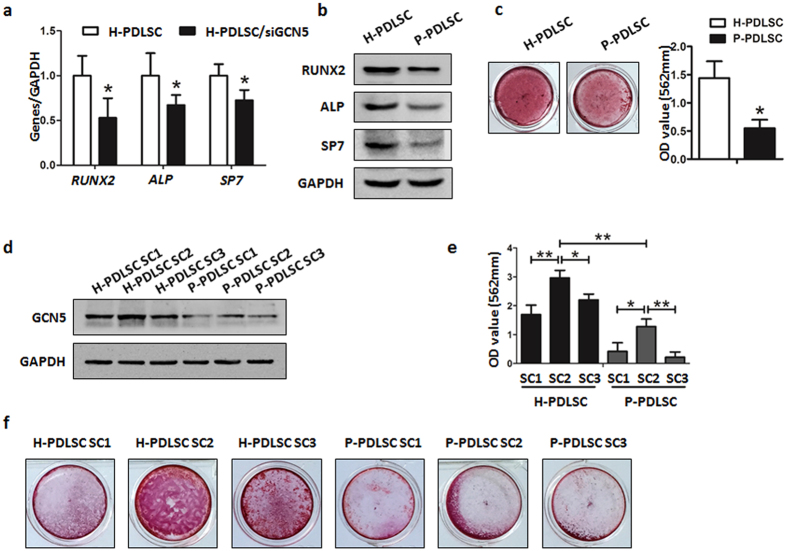
Down-regulated GCN5 expression results in defective osteogenic differentiation of P-PDLSCs. (**a**) Gene expression of Runx2, ALP and SP7 in H-PDLSCs and P-PDLSCs was measured by qRT-PCR after osteogenic induction for 14 days. (**b**) Protein expression of Runx2, ALP and SP7 in H-PDLSCs and P-PDLSCs was measured by western blot after osteogenic induction for 14 days. (**c**) H-PDLSCs and P-PDLSCs were cultured in osteogenic medium and osteogenic differentiation was determined by Alizarin red S staining after 28 days. Alizarin red was then extracted and measured for light absorbance at 562 nm. (**d**) Protein expression of GCN5 in single colony derived H-PDLSCs and P-PDLSCs was measured by western blot. (**e,f**) Single colony derived H-PDLSCs and P-PDLSCs were cultured in osteogenic medium and osteogenic differentiation was determined by Alizarin red S staining after 28 days. Alizarin red was then extracted and measured for light absorbance at 562 nm. Data represent the means ± SD. *p < 0.05, **p < 0.01, ***p < 0.001, (n = 3).

Since GCN5 expression was down-regulated in inflammatory microenvironment, we want to know if GCN5 regulates the osteogenic differentiation of PDLSCs. We induced osteogenic differentiation of H-PDLSCs and P-PDLSCs in osteogenic culture medium, the results showed that P-PDLSCs exhibited decreased osteogenic differentiation after induction. The expression of osteogenic related genes and proteins such as Runx2, ALP and SP-7 were all decreased in P-PDLSCs compared to H-PDLSCs (Fig. 2a,b). Decreased mineralized nodules of P-PDLSCs compared to H-PDLSCs were assessed by Alizarin red S staining (Fig. 2c) after 28 days induction. These results were consistent with our previous study that P-PDLSCs showed defective osteogenic differentiation^9^.

To know the function of GCN5 in cell differentiation, we isolated single colonies of H-PDLSCs and P-PDLSCs. We compared 3 colonies from H-PDLSCs and 3 colonies from P-PDLSCs. We found different GCN5 expression in different colonies from H-PDLSCs and P-PDLSCs as assessed by western blot (Fig. 2d). Colonies from H-PDLSCs showed higher GCN5 expression compared to colonies from P-PDLSCs. Interestingly, single colony 2 (SC2) from H-PDLSCs expressed higher GCN5 compared to the other two colonies from H-PDLSCs (Fig. 2d). SC2 from H-PDLSCs showed more mineralized nodules formation compared to the other two colonies from H-PDLSCs (Fig. 2e,f). In addition, SC2 from P-PDLSCs also expressed higher GCN5 and formed more mineralized nodules compared to the other two colonies from P-PDLSCs (Fig. 2d–f). Formation of mineralized nodules in SC2 from H-PDLSCs was the highest among the 5 groups by Alizarin red S staining and the GCN5 expression of this colony was also the highest among the 5 groups (Fig. 2d–f). These results indicated that cells derived from clone that has higher expression of GCN5 shows more osteogenic differentiation and down-regulation of GCN5 might result in defective osteogenic differentiation of P-PDLSCs.

**Figure 3:**
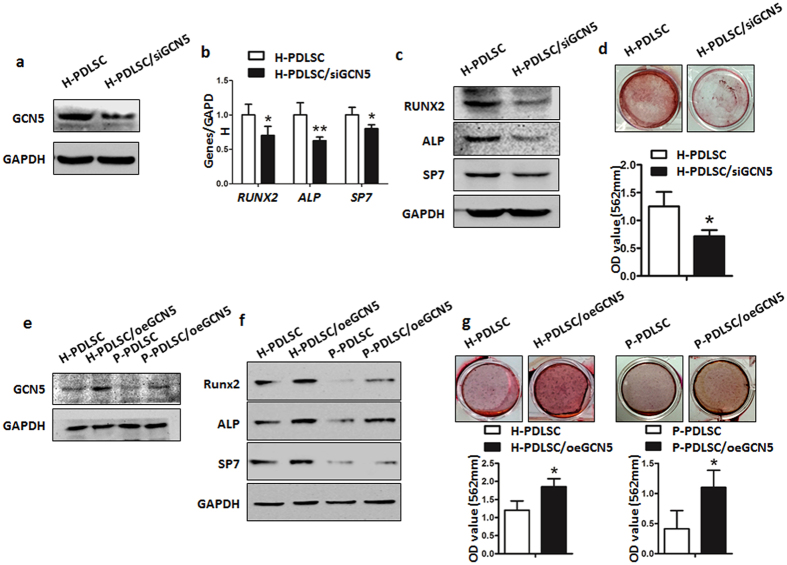
Knockdown of GCN5 leads to defective osteogenic differentiation of H-PDLSCs. (**a**) Western blot analysis showed GCN5 expression of H-PDLSCs and H-PDLSCs/siGCN5. GAPDH was used as the internal control. (**b**) Gene expression of Runx2, ALP and SP7 in H-PDLSCs and H-PDLSCs/siGCN5 was measured by qRT-PCR after osteogenic induction for 14 days. (**c**) Protein expression of Runx2, ALP and SP7 in H-PDLSCs and H-PDLSCs/siGCN5 was measured by western blot after osteogenic induction for 14 days. (**d**) H-PDLSCs and H-PDLSCs/siGCN5 were cultured in osteogenic medium and osteogenic differentiation was determined by Alizarin red S staining after 28 days. (**e**) Western blot analysis showed GCN5 expression of H-PDLSCs, H-PDLSCs/oeGCN5, P-PDLSCs and P-PDLSCs/oeGCN5. GAPDH was used as the internal control. (**f**) Protein expression of Runx2, ALP and SP7 in H-PDLSCs, H-PDLSCs/oeGCN5, P-PDLSCs and P-PDLSCs/oeGCN5 was measured by western blot after osteogenic induction for 14 days. (**g**) H-PDLSCs, H-PDLSCs/oeGCN5, P-PDLSCs and P-PDLSCs/oeGCN5 were cultured in osteogenic medium and osteogenic differentiation was determined by Alizarin red S staining after 28 days. Data represent the means ± SD. *p < 0.05, **p < 0.01, ***p < 0.001, (n = 3).

Since higher GCN5 expression of single colony cells showed higher osteogenic differentiation, we explored whether it played a role in osteogenic differentiation of PDLSCs. We knocked down GCN5 in H-PDLSCs by siRNA (Fig. 3a). After osteogenic induction, we found that GCN5 knockdown reduced the expression of osteogenic differentiation related genes and proteins of RUNX2, ALP and SP7 (Fig. 3b,c). After knockdown of GCN5, formation of mineralized nodules were reduced after prolonged treatment with inducing media for 4 weeks (Fig. 3d). We also over-expressed GCN5 in H-PDLSCs and P-PDLSCs (H-PDLSCs/oeGCN5 and P-PDLSCs/oeGCN5), western blot analysis showed higher GCN5 expression in H-PDLSCs/oeGCN5 compared to H-PDLSCs after transfection (Fig. 3e). The expression of GCN5 was also increased in P-PDLSCs/oeGCN5 compared to P-PDLSCs (Fig. 3e). After osteogenic induction, the results showed higher expression of RUNX2, ALP, SP7 and more mineralized nodules in H-PDLSCs/oeGCN5 compared to H-PDLSCs (Fig. 3f,g). In addition, P-PDLSCs/oeGCN5 also expressed higher RUNX2, ALP, SP7 and formed more mineralized nodules compared to P-PDLSCs after osteogenic induction (Fig. 3f,g).

### GCN5 targets DKK1 to regulate osteogenic differentiation of PDLSCs

**Figure 4:**
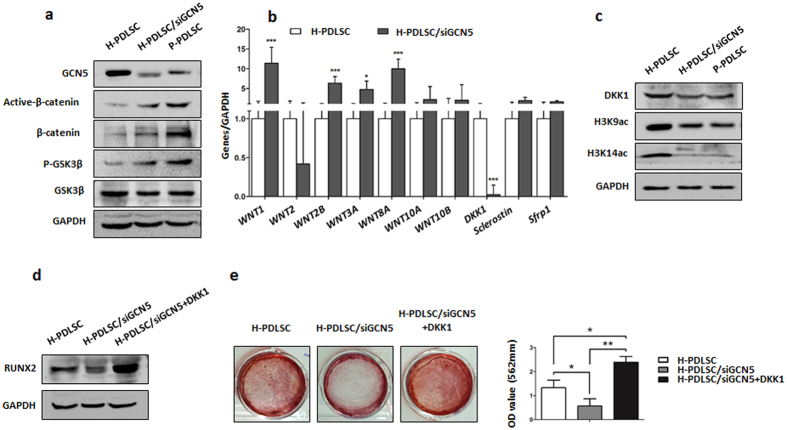
GCN5 targets DKK1 to regulate osteogenic differentiation of PDLSCs. (**a**) Western blot analysis showed expression of GCN5, Active-β-catenin, β-catenin, P-GSK3β, GSK3β of H-PDLSCs, H-PDLSCs/siGCN5 and P-PDLSCs. GAPDH was used as the internal control. (**b**) Gene expression of Wnt ligands and antagonists in H-PDLSCs and H-PDLSCs/siGCN5. Relative gene expression was determined based on the threshold cycle (CT) values. (**c**) Expression of DKK1, H3K9ac and H3K14ac of H-PDLSCs, H-PDLSCs/siGCN5 and P-PDLSCs was determined by western blot. (**d**) Protein expression of Runx2 in H-PDLSCs, H-PDLSCs/siGCN5 and H-PDLSCs/siGCN5+DKK1 was measured by western blot after osteogenic induction for 14 days. (**e**) H-PDLSCs, H-PDLSCs/siGCN5 and H-PDLSCs/siGCN5+DKK1 were cultured in osteogenic medium and osteogenic differentiation was determined by Alizarin red S staining after 28 days. Data represent the means ± SD. *p < 0.05, **p < 0.01, ***p < 0.001, (n = 3).

Our previous study showed that Wnt/β-catenin pathway played an important role in regulating osteogenic differentiation of PDLSCs. Activation of Wnt/β-catenin pathway leaded to the decreased osteogenic differentiation of P-PDLSCs^10^. In order to explore the mechanism of osteogenic differentiation regulated by GCN5, we tested whether depletion of GCN5 affected Wnt/β-catenin pathway. Interestingly, after knockdown GCN5 of H-PDLSCs by siRNA, the expression of active-β-catenin and β-catenin were all increased compared to H-PDLSCs without treatment. In P-PDLSCs, the expression of active-β-catenin and β-catenin were also increased compared to H-PDLSCs, which is consistent with our previous study^10^. We also examined expression level of GSK3β and p-GSK3β. The result showed higher p-GSK3β expression of H-PDLSCs/siGCN5 and P-PDLSCs compared to H-PDLSCs (Fig. 4a), which indicated that GCN5 might affect the upstream factors of Wnt/β-catenin.

We further profiled the expression levels of Wnt ligands and antagonists in H-PDLSCs and H-PDLSCs/siGCN5. The expression of Wnt ligands, Wnt1, Wnt2B, Wnt3A and Wnt8A, were upregulated of H-PDLSCs/siGCN5 (Fig. 4b). However, only the expression of DKK1 of Wnt antagonists was decreased, while the expression level of sclerostin and sfrp1 didn’t changed significantly after knockdown of GCN5 in H-PDLSCs (Fig. 4b). Since GCN5 has been found to have a preference for lysine 9 and lysine 14 on histone H3^25^,^26^, which are marks for gene activation. We tested the expression of DKK1, H3K9ac and H3K14ac of H-PDLSCs, H-PDLSCs/siGCN5 and P-PDLSCs by western blot. The results showed that expression of DKK1, H3K9ac and H3K14ac of H-PDLSCs/siGCN5 and P-PDLSCs was decreased compared to H-PDLSCs (Fig. 4c), suggesting that GCN5 may acetylate H3K9 and H3K14 of DKK1, as DKK1 was downregulated after knockdown GCN5. Activation of Wnt ligands of Wnt1, Wnt2B, Wnt3A and Wnt8A after knockdown GCN5 might be due to the other function of GCN5.

To examine the role of DKK1 in PDLSCs differentiation of PDLSCs, we added DKK1 recombinant protein to H-PDLSCs/siGCN5 during the osteogenic induction and revealed that supplement of DKK1 significantly increased osteogenic differentiation of H-PDLSCs/siGCN5, as assessed by Alizarin red staining showing increased mineralized nodule formation and immunoblotting analysis showing increased expression of the osteogenic markers RUNX2 (Fig. 4d,e). These results suggested that GCN5 targeted DKK1 to regulate osteogenic differentiation of PDLSCs.

### GCN5 acetylates DKK1 through H3K9ac and H3K14ac

**Figure 5:**
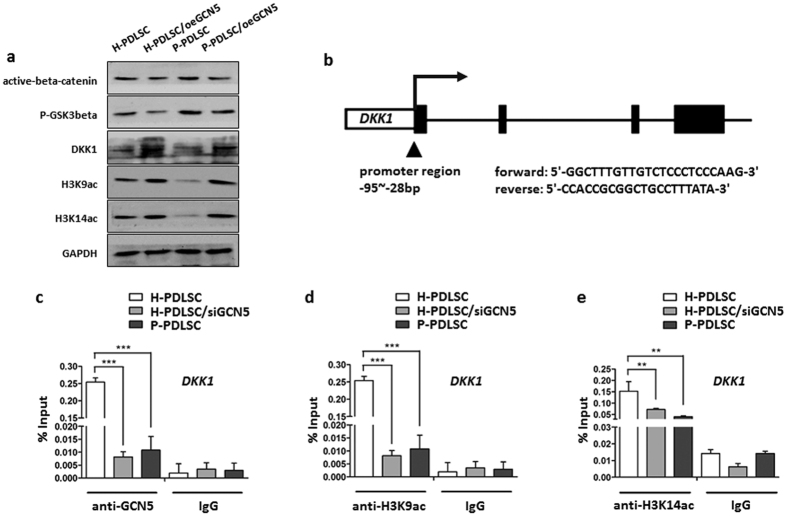
GCN5 acetylates DKK1 through H3K9ac and H3K14ac. (**a**) Protein expression of Active-β-catenin, P-GSK3β, DKK1, H3K9ac and H3K14ac in H-PDLSCs, H-PDLSCs/oeGCN5, P-PDLSCs and P-PDLSCs/oeGCN5. GAPDH was used as the internal control. (**b,c**) Downregulated expression of GCN5 reduced GCN5 binding to the promoter of DKK1 in H-PDLSCs/siGCN5 and P-PDLSCs. The promoters of DKK1 were ChIP-ed with anti-GCN5 antibody or IgG control. (**d,e**) Downregulated expression of GCN5 in H-PDLSCs/siGCN5 and P-PDLSCs decreased H3K9ac and H3K14ac levels at the promoters of DKK1. Data represent the means ± SD. **p < 0.01, ***p < 0.001, (n = 3).

To further examine if overexpression of GCN5 could increase DKK1 acetylation and inhibit Wnt/β-catenin pathway, we compared the expression of active-β-catenin, p-GSK3β, DKK1, H3K9ac and H3K14ac in H-PDLSCs, H-PDLSCs/oeGCN5, P-PDLSCs and P-PDLSCs/oeGCN5 by western blot. The results showed lower expression of active-β-catenin and p-GSK3β in H-PDLSCs/oeGCN5 compared to H-PDLSCs (Fig. 5a). Western blot analysis also showed lower expression of active-β-catenin and p-GSK3β in P-PDLSCs/oeGCN5 compared to P-PDLSCs (Fig. 5a). The expression of DKK1, H3K9ac and H3K14ac was elevated in H-PDLSCs/oeGCN5 and P-PDLSCs/oeGCN5 compared to H-PDLSCs and P-PDLSCs, respectively (Fig. 5a).

In order to examine whether GCN5 regulated osteogenic differentiation of PDLSCs by acetylating H3K9 and H3K14 of DKK1, we performed ChIP assay to assess the changes in histone acetylation status at the promoter region of DKK1. Indeed, we found that GCN5 bound to the promoter region of DKK1. GCN5 occupancy on the promoter of DKK1 was reduced in PDLSCs/siGCN5 and P-PDLSCs compared to H-PDLSCs (Fig. 5b,c). Consistently, decreased binding of GCN5 of PDLSCs/siGCN5 and P-PDLSCs at the promoter region was associated with decreased occurrence of it substrates, H3K9ac and H3K14ac (Fig. 5d,e). The levels of H3K9ac and H3K14ac of PDLSCs/siGCN5 and P-PDLSCs were reduced at the DKK1 promoter compared to H-PDLSCs, which is a hallmark of DKK1 gene silencing.

### Aspirin rescues the periodontitis of LPS-induced periodontitis rats through upregulating GCN5

**Figure 6:**
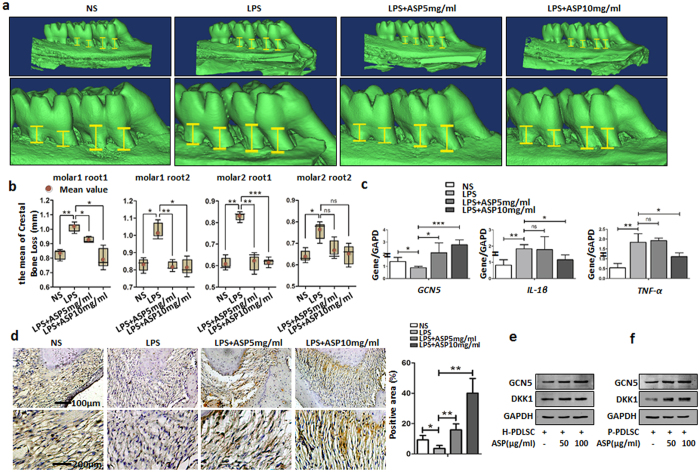
Aspirin rescues the periodontitis of LPS-induced periodontitis rats through upregulating GCN5 expression. (**a**) Alveolar bone loss determination of maxillary molars. Four sites for two molars (one site for each root of one tooth) were analyzed morphometrically. (**b**) Mean alveolar bone loss of two molars. (**c**) Gene expression of GCN5, IL-1β and TNF-α in periodontal tissue was determined by qRT-PCR. (**d**) GCN5 positive staining were showed in the periodontal ligament and its expression in the LPS group was significantly reduced compared to that of the control group. After aspirin (5 and 10 mg/ml) administration, the expression of GCN5 was greatly elevated and was comparable to that of the control group. Integrated intensity of GCN5 positive staining was measured by Image-Pro Plus 6.0. Scale bar: 100 μm, 200 μm. (**e,f**) Western blot showed expression of GCN5 and DKK1 of H-PDLSCs and P-PDLSCs with or without aspirin treatment, respectively. Data represent the means ± SD. *p < 0.05, **p < 0.01, ***p < 0.001, (n = 3).

Since aspirin is widely used for inhibiting inflammation, we tested if aspirin can be used to inhibit periodontitis. We used 5 mg/ml and 10 mg/ml aspirin to treat the periodontitis rat. We found that, in LPS group, the distance from the cemento-enamel junction (CEJ) to the alveolar bone crest at nearly all the 4 sites we tested was the highest (Fig. 6b), which meant that the bone loss in this group was the greatest. With the administration of aspirin, the alveolar bone loss of the first and second maxillary molars was obviously reduced (Fig. 6a), as the distance from the CEJ to the alveolar bone crest was reduced (Fig. 6b) significantly. Statistically analysis of the average bone loss for the 4 sites in each group also proved that aspirin significantly reversed the bone loss caused by LPS (Fig. 6a,b). Further, we tested the expression of IL-1β, TNF-α and GCN5 in periodontal tissue. The result showed that IL-1β and TNF-α were upregulated in LPS-induced periodontitis rats (Fig. 6c). GCN5 was downregulated in LPS-induced periodontitis rats (Fig. 6c). With the injection of aspirin, IL-1β and TNF-α were decreased and GCN5 expression was increased in periodontal tissue. We also measured the expression of GCN5 in periodontal tissue by immunohistochemistry staining. The results showed lower expression of GCN5 in LPS-induced periodontitis rats (Fig. 6d). With the injection of aspirin, the expression of GCN5 were increased in periodontal tissue (Fig. 6d).

In addition, the results showed that aspirin treatment increased the expression of GCN5 and DKK1 both in H-PDLSCs and P-PDLSCs (Fig. 6e,f), which indicated that aspirin might regulate osteogenic differentiation through GCN5. We also found that aspirin treatment increased osteogenic differentiation of H-PDLSCs and P-PDLSCs (Supplementary Fig. S2). Interestingly, aspirin didn’t increase the osteogenic differentiation of H-PDLSCs after knockdown of GCN5 (Supplementary Fig. S3). These results suggested that aspirin could be used to inhibit periodontitis formation through upregulating GCN5 expression.

## Discussion

MSCs possess the potential to differentiate into osteoblast and maintain the bone homeostasis. PDLSCs are a group of tissue specific MSCs derived from periodontal ligament and maintain the homeostasis of periodontal tissue. However, PDLSCs from periodontitis patients exhibited defective osteogeneic differentiation ability compared to those from healthy individuals. How osteogenic differentiation of PDLSCs is regulated in inflammatory microenvironments still need to be identified. In this study, we found that expression of a HAT, GCN5, in P-PDLSCs was reduced in inflammatory microenvironment. Most importantly, GCN5 were documented to play a critical role in osteogenic differentiation of PDLSCs by acetylation of H3K9 and H3K14 on DKK1, which inhibits Wnt/β-catenin pathway and promotes osteogenic differentiation of PDLSCs. Interestingly, we also found that aspirin inhibited the periodontitis development of LPS injected rats through elevating GCN5 expression and promoted osteogenic differentiation of PDLSCs. Our results provide the first demonstration that histone acetylation controls osteogenic differentiation of MSCs in inflammatory microenvironment.

Epigenetic marks change to reflect the activation and repression of genes without changes in the underlying DNA sequence^27^,^28^. Such induced epigenetic changes by internal or external environmental factors can be inherited during cell passages, resulting in permanent maintenance of the acquired phenotype. In this study, we compared the expression levels of HATs in PDLSCs from healthy individuals and periodontitis patients (data not shown) and found that GCN5 was downregulated in P-PDLSCs after several passages. There are a lot of inflammatory cytokines involved in pathology of periodontitis. Due to our previous study, we found higher expression of LPS, TNF-α and IL-1β in diseased periodontal tissue^6^,^9^. Thus, we used LPS, TNF-α and IL-1β alone or in combination to treat PDLSCs, which mimicked the PDLSCs in inflammatory microenvironment. The results showed that *in vitro* mimicked inflammatory microenvironment also decreased the GCN5 expression. After passing PDLSCs to passage 4 and passage 6, downregulation of GCN5 was still maintained. Our results suggest that inflammatory microenvironment change GCN5 expression, which may change the histone acetylation of PDLSCs and thus the differentiation of PDLSCs.

Recently, elevating evidences suggest MSCs differentiation is regulated by histone acetylation^29^,^30^. H3K9ac and H3K14ac marks are generally associated with gene activation. Increase of H3K9ac marks was reported of activating osteogenic differentiation related genes. Recently, Fu *et al*. found that Histone deacetylase 8 (HDAC8) suppresses osteogenic differentiation of MSCs by inhibiting H3K9ac. Interestingly, they found HDAC8 directly binds to Runx2, and inactivation of HDAC8 increased the level of H3K9ac and promotes osteogenic differentiation of MSCs^31^. The creation of conditional Gcn5^flox^ allele results in rib and vertebral malformations consistent with anterior transformations of specific thoracic and lumbar skeletal segments^32^, which indicates that GCN5 may regulate the osteogenesis during development. GCN5 has been found to have a preference for H3K9 and H3K14 acetylation. In our study, we found that reduced expression of GCN5 in P-PDLSC impaired its osteogenic differentiation. In addition, inactivation of GCN5 also reduced osteogenic differentiation of H-PDLSCs. Mechanistically, inactivation of GCN5 activated Wnt/β-catenin pathway and inhibited osteogenic differentiation of PDLSCs. Activated Wnt/β-catenin of PDLSCs impairing its osteogenic differentiation was well documented in our previous study^10^. In this study, we found that inactivation of GCN5 decreased the expression of DKK1, which is the inhibitor of Wnt/β-catenin pathway. GCN5 directly bound to DKK1 promoter region and promoted its expression by increased the level of H3K9ac and H3K14ac.

Periodontitis, an inflammatory bone disease, is associated with osteogenic deficiency of PDLSCs and bone loss^6^,^9^. Our results revealed a significant lower expression of GCN5 in LPS induced periodontitis rat. This result indicates that decreased GCN5 levels in inflammation microenvironment causes bone loss of periodontitis by influencing the differentiation of PDLSCs. Aspirin is a widely used nonsteroidal anti-inflammatory agent. It is previous reported capable of elevating osteogenesis of MSCs^23^. Based on these reasons, we wonder if aspirin is capable of inhibiting alveolar bone loss in LPS-induce periodontitis rat through upregulating the GCN5 expression in periodontal tissue. We found that aspirin treatment inhibiting bone loss in LPS-induce periodontitis rat. Moreover, inflammation factor IL-1β and TNFα in periodontal tissue were reduced after aspirin treatment. Most importantly, aspirin treatment increased the expression of GCN5 in periodontal tissue. These results suggested that aspirin inhibited inflammation and increased GCN5 expression in inflammatory microenvironments. Interestingly, aspirin also increased the expression of GCN5 and DKK1 in PDLSCs and promoted the osteogenesis of H-PDLSCs and P-PDLSCs. These results suggest GCN5 could be upregulated by aspirin to modulate osteogenic differentiation of PDLSCs, thereby holding a promising potential as a therapeutic target for periodontitis treatment. In our study, GCN5 may also regulate the osteogenic differentiation of MSCs in the alveolar bone and influence periodontal regeneration. However, the effect of GCN5 in osteogenic differentiation of MSCs in the alveolar bone and the detailed mechanism of aspirin upregulating GCN5 expression still need further study.

In summary, our study has provided the new insight into deficiency of stem cells in inflammatory microenvironment, revealing that histone acetylation controls osteogenic differentiation of MSCs in such microenvironment. Decreased expression of GCN5 impaired osteogenic differentiation of PDLSCs. Mechanistically, GCN5 activated DKK1 expression by acetylation of H3K9 and H3K14, and thus inhibiting Wnt/β-catenin pathway of PDLSCs. In addition, aspirin inhibited the inflammation and prevented the development of periodontitis in LPS injected rat through elevating GCN5 expression.

## Methods

### Cell culture and identification of stem cells

Healthy human teeth samples were collected from individuals (n = 5, aged from 16 to 30 years) extracted for orthodontic reasons. Adolescent donors were examined on defined variables for clinically healthy periodontal tissues with the absence of bleeding on probing, probing depth <4 mm and loss of attachment level <3 mm. Teeth affected by periodontitis were collected from periodontitis patients, (n = 5, aged 30–45 years). The clinical diagnosis of chronic periodontal disease was based on the visual and radiographic assessment of the periodontal tissues and on measurements of the space between the tooth and gum. The patients diagnosed with periodontitis were defined as alveolar bone loss (2/3) and more than 1 pocket (depth 5 mm). The subjects engaged in this study had no history of systemic diseases, smoking or taking special medicine over the past half year. Written informed consent was provided by all participants, and ethical approval had been obtained from the Ethics Committee of the School of Stomatology, the Fourth Military Medical University. All the methods in the study were carried out in accordance with the approved guidelines.

PDLSCs were isolated and cultured as we previously described^33^. Briefly, after separation the middle third of the root surface, the periodontal ligament (PDL) was enzymatically digested with type I collagenase (0.66 mg/ml; Sigma, St Louis, MO, USA) for 1 hrs (suspended every 10 mins), and then cultured in α-MEM (Invitrogen, Carlsbad, CA, USA) supplemented with 10% fetal bovine serum (FBS; Thermo Electron, Melbourne, Australia), 0.292 mg/ml glutamine (Invitrogen, Carlsbad, CA, USA), 100 U/ml penicillin, and 100 mg/ml streptomycin in 6-well culture dishes (Costar, USA) at 37 °C in a humidified atmosphere of 5% CO_2_ and 95% air. After 2 weeks in culture, cells from PDL became subconfluent. To obtain homogeneous populations of PDLSCs, single cell-derived colony cultures were obtained using the limiting dilution technique. Single-cell derived colonies were used in this study after 2–4 passages. For each experiment, PDLSCs in the same passage were used.

### Flowcytometric characterization of surface markers expression pattern on PDLSCs

PDLSCs were stained with antibodies for stem cell surface markers and analyzed by flow cytometry as described previously^34^. Briefly, to identify the phenotypes of PDLSCs, 5 × 10^5^ cells at the passage 3 were incubated with phycoerythrin (PE) conjugated monoclonal antibodies for human CD146, CD34 (Biolegend, San Diego, CA, USA), CD90, CD14 (eBioscience, San Diego, CA, USA) and fluorescein isothiocynante (FITC) conjugated monoclonal antibodies for STRO-1, CD105 (eBioscience, San Diego, CA, USA) as the manufacturer’s instructions. The incubation procedure was carried out at 4 °C away from light for 1 hour. After washing with PBS, cells were subjected to flow cytometric analysis (Beckman Coulter, Fullerton, CA, USA).

### Osteogenic differentiation assays

To characterize and compare the lineage differentiation potential of H-PDLSCs, P-PDLSCs and H-PDLSCs with GCN5siRNA treatment, cells were subjected to osteogenic induction. 2 × 10^5^ cells per well were maintained in 6-well plates for 24 h and subsequently incubated with osteogenic medium (100 nM dexamethasone, 50 mg/ml ascorbic acid, and 5 mM b-glycerophosphate; (Sigma, St Louis, MO, USA) for 14 to 28 days according to the manufacturer’s instructions. To assess osteogenic differentiation, Real-time polymerase chain reaction analyses and Western blot analyses were performed after 14 d in culture. To assess calcium nodules deposit, cells were fixed with 75% ethanol and stained with 2% alizarin red (Sigma, St Louis, MO, USA) after 21 d in culture. For Alizarin red quantification, 1 ml of 10% cetylpyridinium chloride was added to each well. Light absorbance of the extracted dye was measured at 562 nm.

### Small interfering RNA and transfection assays

PDLSCs were grown to 80% confluence followed by serum starvation for 12 h. siRNA against human GCN5 and negative control (sequences not provided from Ribobio Co., China) were transfected into cells at a final concentration of 50 nM using the Lipo2000 (Invitrogen, Carlsbad, CA, USA) according to the manufacturer’s instructions. After transfection, the cells were harvested at 48 h for RNA or protein extraction. Transfection medium were removed and replaced with osteogenic induction medium for the following experiments.

### Plasmid of GFP-GCN5 and transfection assays

PDLSCs were grown to 80% confluence followed by serum starvation for 12 h. GFP-GCN5 was a gift from Kyle Miller (Addgene plasmid #65386), which was processed by the manufacture at Addgene. Briefly, GFP-GCN5 was amplified in LB medium with Amp overnight, then extracted using Endo-free Plasmid Mini Lit I (OMEGA bio-tech, India). After that, GFP-GCN5 were transfected into cells at 500 ng with Lipo2000 (Invitrogen, Carlsbad, CA, USA) according to the manufacturer’s instructions. After transfection, the cells were harvested at 48 h for protein extraction. Transfection medium were removed and replaced with osteogenic induction medium for the following experiments.

### LPS, TNF-α, IL-1β, DKK1 and Aspirin treatment

PDLSCs of passage 2 were cultured with inflammatory factors as following described: α-MEM, α-MEM with 10 μg/ml *E. Coli* LPS (#L3755, Sigma, St Louis, MO, USA)^33^, α-MEM with 10 ng/ml TNF-α (#300-01A, Pepro-Tech, USA)^6^, α-MEM with 5 ng/ml IL-1β (#200-01B, Pepro-Tech, USA)^6^ and α-MEM with combination (LPS (10 μg/ml), TNF-α (10 ng/ml) and IL-1β (5 ng/ml)). PDLSCs were treated with inflammatory factors for 14 days and then harvested for following tests. Meanwhile, the same cells after these treatment were passed to passage 4 and 6. PDLSCs were harvested for following tests when cells reaching 80% confluence.

To study osteogenic differentiation with siGCN5 and DKK1, 2 × 10^5^ cells per well were seeded to a 6-well plate. After PDLSCs were interfered by siGCN5 for 48 h, conditioned osteogenic medium was changed with or without DKK1 (100 ng/ml, Pepro-Tech, USA). After 14 days culture, cells were harvested and subjected to western blot analyses as previously described for GCN5 and Runx2. Besides, osteogenic differentiation was determined by alizarin red staining as above mentioned.

Aspirin (Sigma, St Louis, MO, USA) at 50 μg/ml^23^ and 100 μ/ml was added to the culturing medium of PDLSCs when they were reaching 80% confluence. After 48 h aspirin treatment, cells were harvested to measure the expression of GCN5 and DKK1 or changed with osteogenic medium for western blot analyses, alizarin red staining as above mentioned.

### Total RNA extraction and quantitative RT-PCR

Total cellular RNA was extracted by TRIzol reagent (Invitrogen, Carlsbad, CA, USA) according to the manufacturer’s instructions. Isolated total RNA was then subjected to reverse transcription using OligodT primer and PrimeScript^®^ RTase (Takara, Dalian, China) according to the manufacturer’s instructions. Quantitative RT-PCR (qRT-PCR) was performed with SYBR^®^ Premix Ex Taq™ II (Takara, Dalian, China) using the C1000TM Thermal Cycler (Bio-Rad, Hercules, CA, USA). The expression levels of the target genes were normalized to that of the housekeeping gene GAPDH. The sequences of primers used are shown in Supplementary Table S1.

### Protein isolation and western blot analysis

Total proteins were extracted with lysis buffer (10 mM Tris–HCl, 1 mM ethylenediaminetetraacetic acid (EDTA), 1% sodium dodecyl sulfate, 1% Nonidet P-40, 1:100 proteinase inhibitor cocktail, 50 mM β-glycerophosphate, 50 mM sodium fluoride) (Beyotime, Shanghai, China). The protein concentration was determined with a protein assay kit (Beyotime) following the manufacturer’s instructions. Aliquots of 40 to 50 μg per sample were separated by 10% sodium dodecyl sulfate-polyacrylamide gel electrophoresis (SDS-PAGE), transferred to polyvinylidene fluoride (PVDF) membranes (Millipore, Billerica, MA, USA) and blocked with 5% bovine serum albumin (BSA) in PBST (PBS with 0.1% Tween), then incubated with the following primary antibodies overnight: anti-ALP (#ab65834), anti-SP7 (#ab187158), anti-GSK3β (#ab2602), anti-β-Catenin (#ab32572), anti-DKK1 (#ab61275), anti-H3K9Ac (#ab10812), anti-H3K14Ac (#ab52946) (Abcam, Cambridge, UK), anti-GCN5 (#sc-365321, Santa Cruz Biotechnology, CA, USA), anti-RUNX2 (#12556), anti-phospho-GSK3β (#9323) (Cell Signaling Technology, Beverly, MA, USA), anti-active-β-Catenin (#05-665, Millipore, Billerica, MA, USA), anti-GAPDH (#CW0100, CWBIO, China). Then, the membranes were incubated with horseradish peroxidase-conjugated secondary antibody (Boster, Wuhan, China). The blots were visualized using an enhanced chemiluminescence kit (Amersham Biosciences, Piscataway, NJ, USA) according to the manufacturer’s instructions.

### Chromatin immunoprecipitation

The ChIP analysis was performed using EZ-ChIP (Millipore, Billerica, MA, USA). Normal Rabbit IgG (Millipore, Billerica, MA, USA) was used as a negative control. Briefly, PDLSCs, PDLSC/siGCN5 or P-PDLSCs were cross-linked in 1% formaldehyde for 10 min and lysed in SDS lysis buffer and then sonicated to shear DNA. Lysates after diluted with ChIP dilution buffer were immunoprecipitated with rabbit IgG and anti-GCN5 (#sc-365321, Santa Cruz Biotechnology, CA, USA), anti-H3K9Ac (#ab10812, Abcam, Cambridge, UK), anti-H3K14Ac (ab10812, Abcam, Cambridge, UK), overnight at 4 °C. Antibody–chromatin complexes were precipitated with ChIP blocked protein G agarose for 2 h at 4 °C, and then washed andeluted. After reverse crosslink of protein-DNA complexes, DNA was purified using spin columns (Takara, Dalian, China) and analyzed by quantitative RT-PCR. The primers of promoter of DKK1 used were as follows: forward, 5′-GGCTTTGTTGTCTCCCTCCCAAG-3′, and reverse, 5′-CCACCGCGGCTGCCTTTATA-3′^35^.

### Induction of experimental periodontitis of rat

All animal procedures were performed according to the guidelines of the Animal Care Committee of the Fourth Military Medical University, Xi’an, China. 12 adult female Sprague–Dawley rats (SD rats, 8 weeks, 200.7 ± 20.3 g, obtained from the Laboratory Animal Research Center of the Fourth Military Medical University) were used in this protocol. Rats were injected drugs, (saline, LPS 1 mg/ml and Aspirin 5 mg/ml or 10 mg/ml), into the maxillary palatal gingival between the first and second upper molars. The injections were repeated every other day for one week.

12 rats were randomly distributed into 4 groups of 3 rats each: (1) saline: gingiva was injected with 20 μl of saline; (2) LPS: gingiva was injected with 10 μl of LPS and then 10 μl of saline; (3) LPS + Aspirin (5 mg/ml): gingiva was injected with 10 μl of LPS and then 10 μl of Aspirin; and (4) LPS + Aspirin (10 mg/ml): gingiva was injected with 10 μl of LPS and then 10 μl of Aspirin. On day 7, all rats were anesthetized and euthanized by exsanguination. The whole head was removed, one side of mandible were scanned and analyzed using a microCT system (Siemens Inveon MicroCT, Munich, Germany). Then, samples were fixed in 4% paraformaldehyde and decalcified with 10% EDTA before paraffin embedding for Immunohistochemistry. The other side of mandible were extracted RNA for quantitative RT-PCR.

### MicroCT analysis

After the mandible were scanned by a microCT system, rebuilt images of bone surface were used to perform three-dimensional histomorphometric analysis with the same density. From rebuilt images, the alveolar bone height was measured at 4 different sites for two molars (1 site for each of 2 roots of one teeth) by recording the distance from the cemento-enamel junction (CEJ) to the alveolar bone crest. The distance were assessed as the mean distance of experiment groups were compared with the LPS group with statistics.

### Immunohistochemistry

To quantify the expression of GCN5 in periodontal ligament tissue, paraffin embedded tissue sections were de-waxed in xylene and rehydrated through graded alcohols to water. Endogenous peroxidase was blocked using 3% H_2_O_2_ for 15 minutes. For antigen retrieval, 0.3% trypsin (Sigma, St Louis, MO, USA) was used for 15 minutes. Sections were blocked with 10% serum for 30 minutes. Slides were incubated with primary antibody anti-GCN5 (#sc-365321, Santa Cruz Biotechnology, CA, USA) for two hours. Goat anti-mouse secondary antibody was applied for one hour at room temperature. Sections were then incubated in strept avidin-biotin complex (SABC) (Boster) for 30 minutes. Diaminobenzidine (DAB) solution was applied for two to five minutes and development of the color reaction was monitored microscopically. Slides were counterstained with hematoxylin, dehydrated, cleared and then mounted. The slides were observed under a light microscope (BX-51, Olympus, Japan), and images were acquired using a CDD camera. Quantification of GCN5-positive staining was carried out using the software of Image-Pro Plus 6.0.

### Statistical analyses

All experiments were repeated at least three times and data are presented as mean ± SD. Comparisons between two groups were performed using independent unpaired two-tailed Student’s t-test, and the comparisons between more than two groups were analyzed using one-way ANOVA with the Bonferroni adjustment. SPSS 13.0 (IBM, Armonk, NY) software was utilized and *P* values less than 0.05 were considered statistically significant.

## Additional Information

**How to cite this article**: Li, B. *et al*. GCN5 modulates osteogenic differentiation of periodontal ligament stem cells through DKK1 acetylation in inflammatory microenvironment. *Sci. Rep.* **6**, 26542; doi: 10.1038/srep26542 (2016).

## Supplementary Material

Supplementary Information
