# Supplementary material for: GCN5 modulates osteogenic differentiation of periodontal ligament stem cells through DKK1 acetylation in inflammatory microenvironment
Source: Sci Rep. 2016 May 24;6:26542. doi: 10.1038/srep26542 (PMC4877597; doi:10.1038/srep26542)
Supplement: Supplementary Information [file srep26542-s1.pdf]

GCN5 modulates osteogenic differentiation of periodontal ligament stem cells through DKK1 acetylation in inflammatory microenvironment

Bei Li[1,2a], Jin Sun[1,3a], Zhiwei Dong [4a], Peng Xue[1,2], Xiaoning He[1,2], Li Liao[1,2], Lin Yuan[3*], Yan Jin[1,2*]

Supplementary Table S1
Primer sequence used for polymerase chain reaction amplifications.

Gene	Primer sequences	
h-GCN5	Forward	5′-CCAAATCAAGTCTCACCCCAGT-3′
	Reverse	5′-GGAAGCGGATGACCTCGTAG-3′
h-Runx2	Forward	5′-CCCGTGGCCTTCAAGGT-3′
	Reverse	5′-ATGACAGTACCGCCCATTGC-3′
h-ALP	Forward	5′-GGACCATTCCCACGTCTTCAC-3′
	Reverse	5′-ATGACAGTACCGCCCATTGC-3′
h-SP7	Forward	5′-GCCAGAAGCTGTGAAACCTC-3′
	Reverse	5′-GCTGCAAGCTCTCCATAACC-3′
h-WNT1	Forward	5′-CGGGCAACAACCAAAGTCG-3′
	Reverse	5′-CGTTCACAATACCCCACCAT-3′
h-WNT2	Forward	5′-ACAGCAGGCCGTGTGTGTGCAA-3′
	Reverse	5′-AGGCAGTCCTGACAGCGCAC-3′
h-WNT2B	Forward	5′-GACCGGGACCACACCGTCTTTGG-3′
	Reverse	5′-GGTGGAGGGTGGAGGAAGGTG-3′
h-WNT3A	Forward	5′-CTCGGATACTTCTTACTCCTCTGC-3′
	Reverse	5′-CCTGGATGCCAATCTTGATG-3′

h-WNT8A	Forward	5′-GATGCCAGAGCCCTGATGAA-3′
	Reverse	5′-GCCTGGTCATACTTGGCCTT-3′
h-WNT10A	Forward	5′-TTCTTCCTACTGCTGCTGGC-3′
	Reverse	5′-TTAGGCACACTGTGTTGGCA-3′
h-WNT10B	Forward	5′-CAACACCGTGTGCTTGACG-3′
	Reverse	5′-CAGCCAGCATGGAGAAGGAA-3′
h-DKK1	Forward	5′-GGGAATTACTGCAAAAATGGAATA-3′
	Reverse	5′-ATGACCGGAGACAAACCAGAAC-3′
h-Sclerostin	Forward	5′-AGCTGGAGAACAACAAGACCA-3′
	Reverse	5′-CATCGGTCACGTAGCGGG-3′
h-Sfrp1	Forward	5′-GATGCAGGAGGCTCAGGTGAT-3′
	Reverse	5′-GCTGGCAACAGGTCAGAACG-3′
r-GCN5	Forward	5′-TCGTCTTCTGTGCTGTCACC-3′
	Reverse	5′-TCGGCGTAGGTGAGGAAGTA-3′
r-TNFα	Forward	5′-GACCCTCACACTCAGATCATCTTCT-3′
	Reverse	5′-TGCTACGACGTGGGCTACG-3′
r-IL-1β	Forward	5′-TGGCAACTGTCCCTGAACTC-3′
	Reverse	5′-GTCGAGATGCTGCTGTGAGA-3′
GAPDH	Forward	5′-CTGCAAGAACAGCATTGCAT-3′
	Reverse	5′-GACCACCTGGTCCTCAGTGT-3′

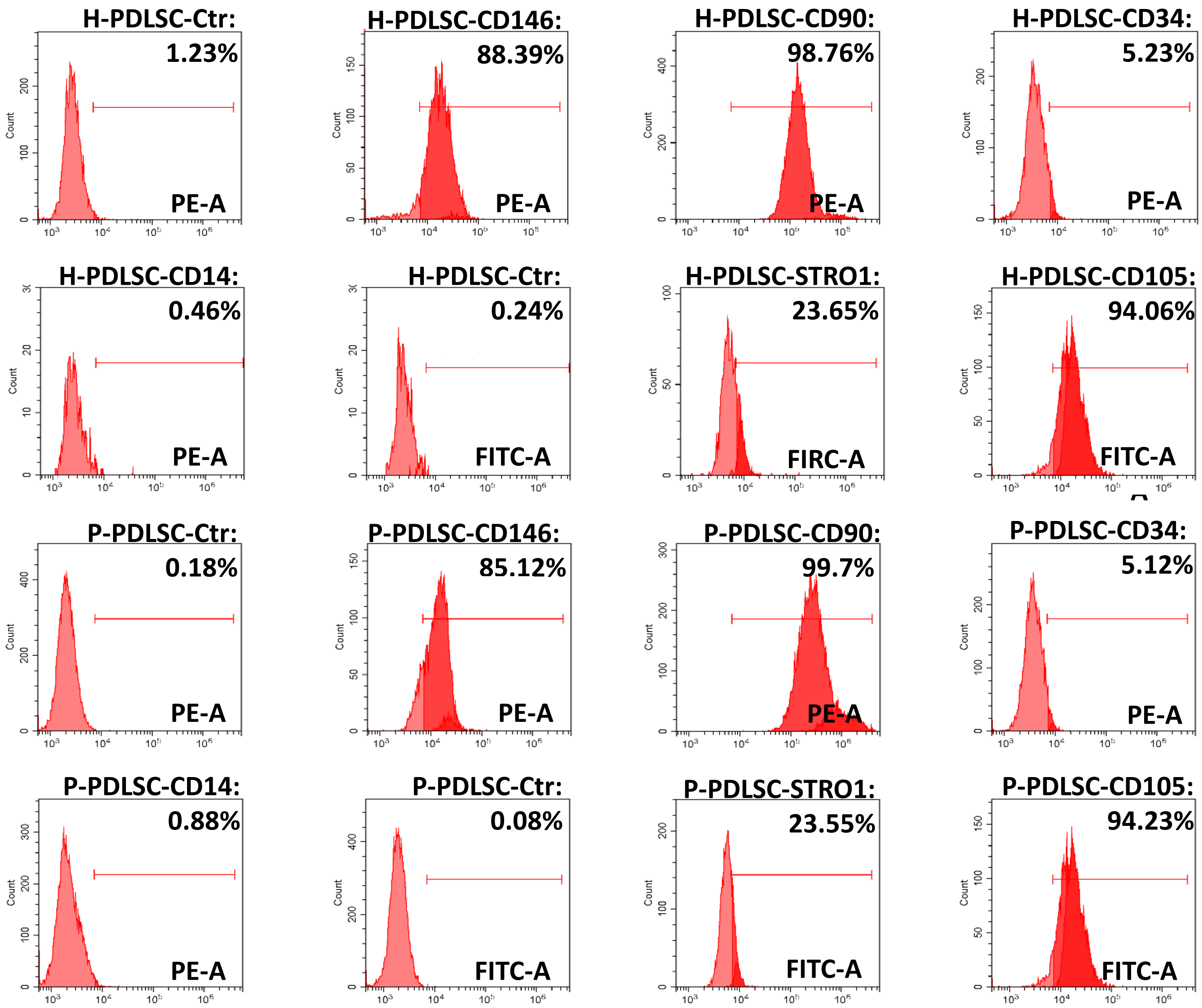

Fig. S1

Supplementary Figure 1: Immunophenotype of PDLSCs.
Immunophenotype analysis of H-PDLSCs and P-PDLSCs from each group was determined by Flow cytometry assay.

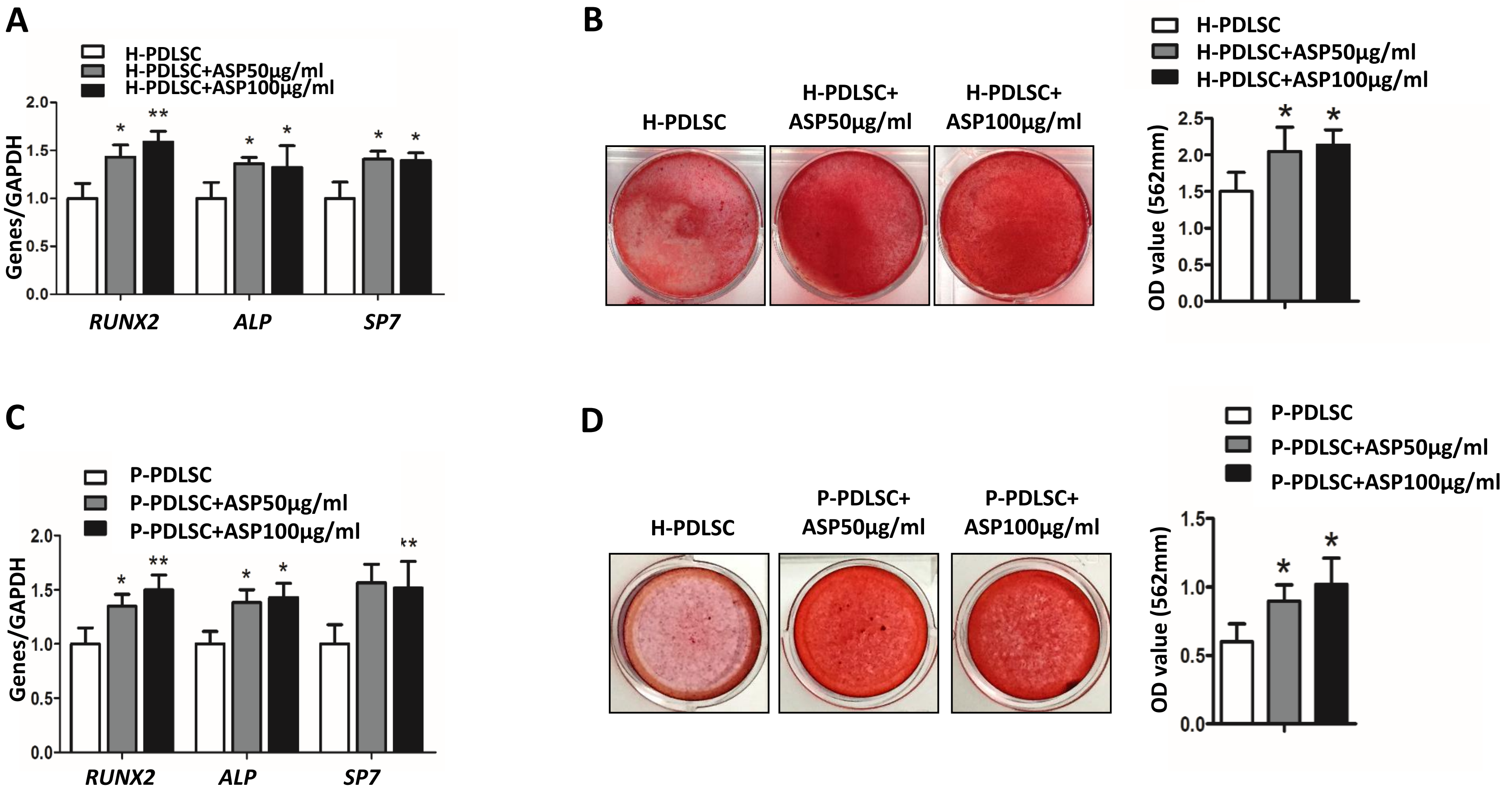

Fig. S2

Supplementary Figure 2: Aspirin promotes osteogenic differentiation of P-PDLSCs.

(A) Gene expression of Runx2, ALP and SP7 in H-PDLSCs and H-PDLSCs with aspirin treatment (50 and 100μg/ml) was measured by qRT-PCR after osteogenic induction for 14 days. (B) H-PDLSCs with or without aspirin treatment were cultured in osteogenic medium and osteogenic differentiation was determined by Alizarin red S staining after 28 days. (C) Gene expression of Runx2, ALP and SP7 in P-PDLSCs and P-PDLSCs with aspirin treatment (50 and 100μg/ml) was measured by qRT-PCR after osteogenic induction for 14 days. (D) P-PDLSCs with or without aspirin treatment were cultured in osteogenic medium and osteogenic differentiation was determined by Alizarin red S staining after 28 days. Data represent the means ± SD. $*p < 0.05$, $** p < 0.01$, $(n = 3)$.

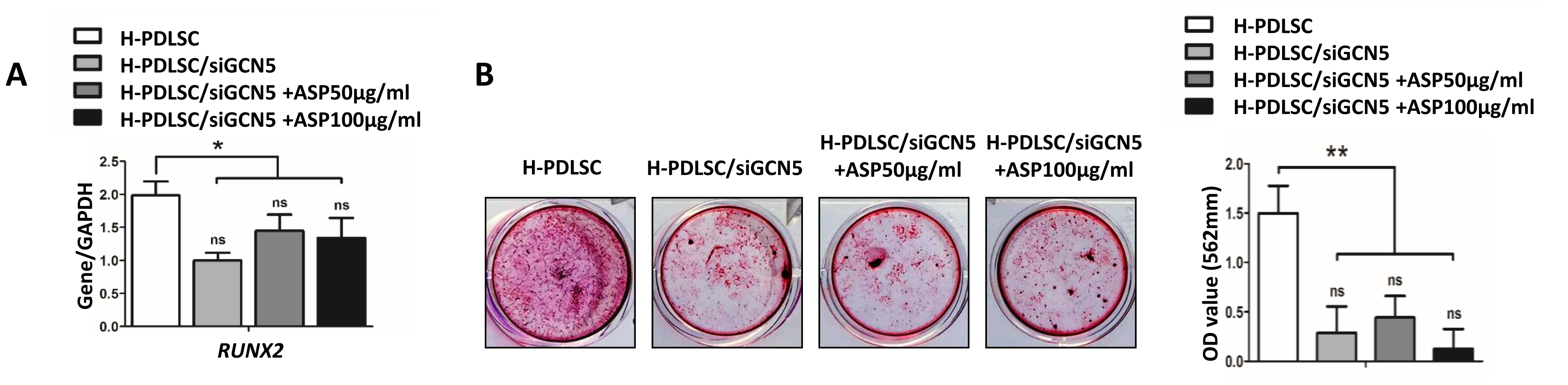

Fig. S3

Supplementary Figure 3: Aspirin fails to increase osteogenic differentiation after knockdown GCN5. (A) Gene expression of Runx2 in H-PDLSCs, H-PDLSCs/siGCN5 and H-PDLSCs/siGCN5 with aspirin treatment (50 and 100μg/ml) was measured by qRT-PCR after osteogenic induction for 14 days. (B) H-PDLSCs, H-PDLSCs/siGCN5 and H-PDLSCs/siGCN5 with aspirin treatment (50 and 100μg/ml) were cultured in osteogenic medium and osteogenic differentiation was determined by Alizarin red S staining after 28 days. Data represent the means $\pm$ SD. $*p < 0.05$, $** p < 0.01$, $(n = 3)$.
